# Ureolytic Rhizobacteria for Cadmium Remediation: Contribution of Bioadsorption and Bioaccumulation During Microbially Induced Carbonate Precipitation in Liquid Medium Assays

**DOI:** 10.1155/ijm/9681937

**Published:** 2026-08-03

**Authors:** Carlos A. Adarme-Duran, Elianna Castillo, Pedro F. B. Brandão

**Affiliations:** ^1^ Universidad Nacional de Colombia-sede Bogotá, Facultad de Ciencias, Instituto de Biotecnología IBUN, Bogotá, Colombia, unal.edu.co; ^2^ Universidad Nacional de Colombia-sede Bogotá, Facultad de Ciencias, Departamento de Química, Grupo de Estudios para la Remediación y Mitigación de Impactos Negativos al Ambiente (GERMINA), Bogotá, Colombia, unal.edu.co

**Keywords:** bioprecipitation, biosorption, Cd, MICP, removal mechanism, ureolytic bacteria

## Abstract

Microbially induced carbonate precipitation (MICP) is a recognized bioremediation strategy for mitigating toxic metal pollution; however, the relative contributions of bioprecipitation, biosorption, and bioaccumulation to cadmium (Cd) removal during MICP remain underexplored. This study assessed Cd immobilization by six rhizosphere ureolytic bacterial strains from different genera in liquid medium and quantified the metal distributed among the bioprecipitated, biosorbed, and bioaccumulated fractions. Minimum inhibitory concentration assays showed Cd tolerance ranging from 90 to 300 mg L^−1^. The strains presented total Cd removal efficiencies of 64.9%–99.8% within 120 h. Notably, Cd removal occurs mainly under 48 h. Biosorption assays revealed substantially lower Cd removal compared with bioprecipitation experiments. Comparative analysis of Cd bioprecipitation and biosorption assays, combined with sequential extraction, demonstrated that bioadsorption plays a significant role in the removal of Cd via MICP. Precipitates characterization further confirmed coprecipitation of Cd with vaterite, a calcium carbonate polymorph. These findings elucidate an important role of biosorption in Cd immobilization during MICP, highlighting the need to evaluate removal mechanisms when assessing bacterial MICP potential, as different mechanisms can lead to distinct long‐term stability outcomes.

## 1. Introduction

Toxic elements in water and soils disrupt ecosystems [1, 2] and pose significant human health risk due to bioaccumulation in food crops [3]. Cadmium (Cd) represents a major issue due to its high mobility, availability, and pronounced toxicity to living organisms [4, 5]. In cacao agriculture, Cd uptake through roots leads to bean contamination, directly impacting chocolate safety [6]. These risks underscore the urgent need for effective soil remediation strategies to mitigate Cd exposure in agricultural soils.

Microbiologically induced carbonate precipitation (MICP) has recently emerged as a bioremediation strategy to treat metal contaminated environments and has gained the attention of the scientific community due to its promising results [7]. Among the metabolic pathways to accomplish MICP, carbonate precipitation by ureolytic bacteria has been widely studied and applied [8, 9]. Briefly, urea hydrolysis by urease produces NH_3_ and NH_2_COOH. The latter is degraded and generates H_2_CO_3_ and further NH_3_, thus increasing pH and shifting H_2_CO_3_ equilibrium favoring the formation of CO_3_^2−^. This carbonate ion can react with divalent ions (e.g. Cd^2+^) to precipitate them and reduce their availability [10].

MICP investigations have typically focused on the search for new isolates with high precipitation performance, optimization of metal removal, and characterization of precipitates [9, 11, 12]. However, most studies report overall metal removal efficiencies without discriminating among the underlying mechanisms responsible for metal immobilization. This limitation arises because commonly used analytical approaches do not differentiate between metal fractions associated with mineral precipitation, surface adsorption, or intracellular accumulation. As a result, the relative contribution of different metal–bacteria interactions (e.g., biosorption and bioaccumulation) during MICP remains poorly understood, particularly for toxic metals such as Cd^2+^.

In a recent study, using the ureolytic bacterium *Pseudochrobactrum* sp. DL‐1, it was shown that bioadsorption and bioaccumulation contribute 36.35% and 3.97%, respectively, to Cd removal from solution by MICP [11]. It has also been reported that a portion of the metals Cd, Cu, Cr, Ni, and Zn, removed by *Providencia rettgeri* QZ2 and *Pseudomonas aeruginosa* QD5 and QZ9 (exhibiting MICP activity), occurs through bioadsorption processes, highlighting that for Ni, only 7% of the removed metal corresponded to bioprecipitation [13]. Additionally, separate bioadsorption and MICP experiments demonstrated that Cd removal was 11% higher in the MICP assay, with *Bacillus* sp. GZ‐22 achieving a removal efficiency of 53.06% [14].

Cd immobilization exhibits strain‐dependent variability, and both biosorption and bioprecipitation may constitute a relevant contribution to overall metal removal. This variability highlights the need for studies that quantify the relative contributions of different removal mechanisms such as bioadsorption and bioaccumulation during MICP assays. Determining the percentage of total Cd removed via each process is essential for understanding how much of the element is bioprecipitated. This is important because it can provide information to understand the long‐term stability of metals immobilized by MICP, which is primarily linked to the low solubility of the resulting carbonate minerals [10].

Based on the available literature and the reported similarity in metal–bacteria interaction behaviors within the same genus, we hypothesize that, in addition to bioprecipitation, a fraction of Cd removal during MICP is attributable to biosorption processes. Thus, the aim of this study was to assess the Cd removal performance of six ureolytic bacterial strains belonging to different genera through bioprecipitation (via MICP) and biosorption experiments. Cd removal from liquid medium was quantified by partitioning the process into bioprecipitation, biosorption, and bioaccumulation components. Three different approaches were employed to determine the precipitated Cd: (i) comparative analysis of bioprecipitation and biosorption assays, (ii) Cd sequential extraction, and (iii) analysis of precipitates using SEM‐EDX, DRX, and atomic absorption spectroscopy. This methodological framework advances the assessment of MICP potential in ureolytic bacteria.

## 2. Material and Methods

### 2.1. Reagents and Bacteria

All chemical reagents used were of analytical grade. Stock solutions of Ca (II) (27 g L^−1^), urea (500 g L^−1^), and Cd (II) (10 g L^−1^) were prepared from CaCl_2_·2H_2_O (PanReac AppliChem, Germany), urea (ChemCruz, United States), and CdCl_2_·H_2_O (Merck, Germany), respectively. The culture media was sterilized by autoclave (121°C, 15 psi, 30 min). Ca, Cd, and urea solutions were sterilized by filtration with 0.22‐*μ*m filters (Sartorius Biolab Products, Germany). Once sterilized, these solutions were added to the autoclaved culture medium under aseptic conditions.

The six ureolytic bacteria exhibiting MICP activity used in this study—*Serratia* sp. 89a, *Comamonas* sp. 76f, *Bacillus* sp. 85d, *Citrobacter* sp. 101a, *Klebsiella* sp. 76b, and *Stenotrophomonas* sp. 67w (GenBank Accession Numbers: PQ497371, PQ497394, PQ497374, PQ497358, PQ497391, and PQ497383, respectively)—were previously isolated from rhizosphere soils of *Theobroma cacao* L. in Santander, Colombia [15, 16]. These strains were maintained in liquid medium (tryptose 5 g L^−1^, peptone 10 g L^−1^, NaCl 5 g L^−1^, urea 20 g L^−1^, and glycerol 20% v/v) at −20°C. For each bacterium, a preinoculum was made in lysogeny broth (LB) medium (tryptose 10 g L^−1^, yeast extract 5 g L^−1^, and NaCl 10 g L^−1^), incubating overnight (30°C, 120 rpm) prior to their experimental use. Then, the optical density at 600 nm (OD_600_, NanoDrop 2000c, Thermo Fisher Scientific, United States) ≈0.3 was adjusted with NaCl (0.85% w/v) and inoculated at 1% (v/v).

### 2.2. Minimum Inhibitory Concentration (MIC)

The MIC was determined from bacterial growth in LB medium with different concentrations of Cd. The preinoculum of each bacterium was inoculated in 10‐mL LB medium with Cd concentrations from 60 to 300 mg L^−1^. The pH of the medium was adjusted to 6.8 with NaOH (1 M). Bacterial growth (OD_600_) was determined after 48 h incubation at 30°C and 120 rpm. The MIC was considered as the concentration at which an OD_600_ ≈ 0 was observed [17]. The assays were performed in triplicate. The uninoculated culture medium was used as a negative control, and the culture medium without Cd, but inoculated with bacteria, was used as a growth positive control.

### 2.3. Cd Removal in Solution by Ureolytic Bacteria

Cd removal experiments were determined in amber flasks with 10‐mL LB medium supplemented with urea (20 g L^−1^), Ca (1.2 g L^−1^), and Cd (60 mg L^−1^), inoculated (1% v/v) with each bacterium. The cultures were incubated at 30°C and 120 rpm, during 120 h. Culture medium aliquots (0.5 mL) were taken every 24 h, centrifuged for 10 min at 8500 rpm, filtered (0.22 *μ*m), and treated with aqua regia (3 HCl: 1 HNO_3_). Quantification of Cd was performed using external calibration on a curve prepared with a Cd standard solution (1000 mg L^−1^, Panreac, Spain) and measured by flame atomic absorption spectroscopy—AAS (CS‐FAAS, ContrAA 700—High‐Resolution Continuum Source, equipped with a short‐arc xenon lamp and a high‐resolution double monochromator, Analytik Jena, Germany). All Cd removal tests were performed in triplicate. The medium without bacteria was used as an abiotic control.

The initial Cd concentration (60 mg L^−1^) used in the experiments was selected considering the levels previously reported in cacao‐producing soils of the study region. Bravo et al. [18] reported Cd concentrations of up to 27 mg kg^−1^ in Santander, Colombia, although values outside the interval of 0.01–30 mg kg^−1^ were excluded from their analysis, suggesting that localized hotspots with higher concentrations may occur. Subsequently, Joya‐Barrero et al. [19] reported Cd concentrations of up to 47 mg kg^–1^ in the same region. Based on these reports and considering that the experiments were conducted in liquid medium, a concentration of 60 mg L^−1^ was selected as a conservative upper‐bound scenario.

### 2.4. Removal of Cd in Bioprecipitation and Biosorption Assays Evaluated Through Sequential Extractions

For the bioprecipitation assay, 10‐mL LB medium supplemented with urea (20 g L^−1^), Ca (1.2 g L^−1^), and Cd (60 mg L^−1^) was inoculated with bacteria. For the biosorption assay, LB medium supplemented with Cd (60 mg L^−1^) was used. Under the latter conditions, without the addition of urea–Ca, the carbonate precipitation is not induced, which is why this experiment is considered a biosorption assay. Both bioprecipitation and biosorption experiments were incubated for 48 h at 30°C and 120 rpm. Bioadsorbed (Fraction F1), bioprecipitated (Fraction F2), and bioaccumulated (Fraction F3) Cd were determined using previously described methodologies [11, 20, 21] in both bioprecipitation and biosorption experiments, through sequential extractions. This methodology is presented in more detail in the supporting material and its Figure S1. In this way, three operationally defined fractions are obtained: the EDTA‐extracted fraction, associated with the metal bioadsorbed on the cell surface; the HCl‐extracted fraction, associated with the bioprecipitated metal; and the aqua regia‐extracted fraction, corresponding to the metal bioaccumulated inside the cell. Uninoculated culture media were used as abiotic control. Additionally, Cd removed in both biosorption and bioprecipitation assays can be used to calculate the metal % bioprecipitated according to Equation (1).

%Cdbioprecipitated=mg Cd removedbioprecipitation essay−mg Cd removedbiosorption assaymg Cd removedbioprecipitation assay×100.

### 2.5. Production and Analysis of Bioprecipitates

To obtain bioprecipitates for analysis, *Bacillus* sp. 85d and *Stenotrophomonas* sp. 67w were incubated for 15 days at 30°C and 120 rpm in 10‐mL LB supplemented with urea (20 g L^−1^), Ca (1.2 g L^−1^), and Cd (60 mg L^−1^). After incubation, the culture medium was shaken and centrifuged for 10 min at 8500 rpm. The supernatant was discarded, and the pellet (biomass + precipitates) was washed with deionized water. To separate the biomass from the precipitates, the pellet was resuspended in deionized water and transferred to a beaker. The suspension was heated in a conventional microwave oven for 1–2 min. Then, the precipitates were allowed to settle. The biomass, having a lower density, remains suspended in the water, and thus the supernatant was discarded. This process was repeated until no turbidity was observed in the supernatant [22]. The precipitates obtained had a brown color (probably due to adhered biomass); for this reason, they were treated with 5 mL of 10% H_2_O_2_ (pH = 8) [23, 24] and washed with deionized water until white precipitates were obtained. The precipitates were dried at 60°C for 48 h and stored in a desiccator.

The identification of the precipitates was performed by x‐ray diffraction (XRD, X‐pert PRO Diffractometer, PANalytical, the Netherlands). The analyses were performed under CuK*α* radiation (*λ* = 1.540598 Å), with a scan from 10° to 90° (2*θ*). Identification was performed by comparison with the ICSD database using X′Pert HighScore Plus software Version 3.0.5 (PANalytical, 2012, Almelo, the Netherlands). Additionally, the morphology of the precipitates was obtained by scanning electron microscopy (SEM) (VEGA3, TESCAN, Czech Republic) using backscattered electron (BSE) and secondary electron (SE) detectors. Elemental analysis of the surface of the precipitates was performed by energy‐dispersive x‐ray spectroscopy (EDX) using an XFlash 410 detector (Bruker, Germany) coupled to the SEM. Finally, to quantify Cd by AAS (as described before in Section 2.3), a portion of the precipitates was dissolved in HCl (1.2 M) and brought to final volume with HNO_3_ (0.5% v/v).

### 2.6. Statistical Analysis

Data analysis was performed using R software Version 4.2.2. RStudio 2022 Version 12.0 + 353. Experiments were performed in triplicate and results are presented as mean ± standard deviation (SD). Error bars in the graphs correspond to SD. Statistically significant differences when comparing biosorption and bioprecipitation assays were determined by *t*‐test.

## 3. Results and Discussion

### 3.1. MIC of Cd

**Figure 1:**
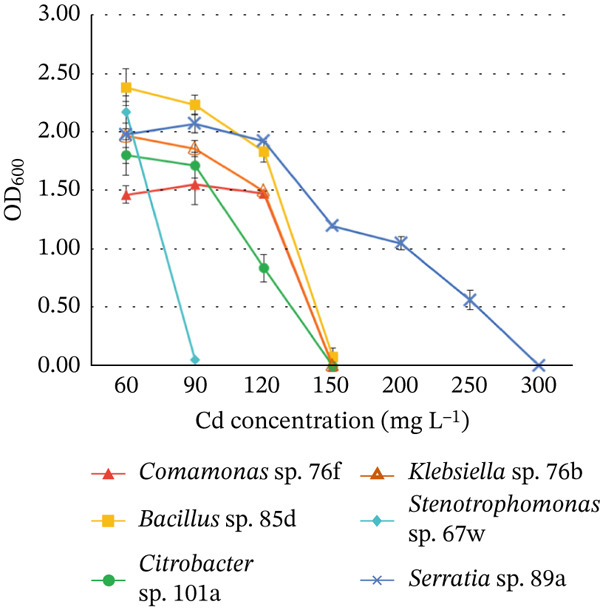
Bacterial growth of ureolytic bacteria at different concentrations of Cd in LB medium, after an incubation period of 48 h at 30°C and 120 rpm. Error bars indicate SD (*n* = 3).

The studied strains exhibited MIC values ranging from 90 to 300 mg L^−1^ according to OD_600_ measurements (Figure 1). *Serratia* sp. 89a showed the highest tolerance to Cd with a value of 300 mg L^−1^, threefold greater than the MIC of *Stenotrophomonas* sp. 67w with the lowest value. MIC values found in this study align with previous reports for cacao soil bacteria [22, 25]. *Serratia* sp. 4.1a and 5b showed complete inhibition of bacterial growth at 4.00 mM (≈450 mg L^−1^) [22], whereas *Burkholderia* sp. NB10 and *P. aeruginosa* NB2 had MICs of 140 mg kg^−1^ [25]. The latter authors found for *P. putida* GB78 a lower MIC of 90 mg kg^−1^.

*Comamonas* sp. 76f showed a tolerance of 150 mg L^−1^, higher than that reported for *Comamonas* sp. A13 and A23 with a MIC of 0.8 mM (≈90 mg L^−1^), both isolated from contaminated rice crop soils [26]. The strain *Bacillus* sp. 85d with a MIC of 150 mg L^−1^ showed a higher tolerance than *Bacillus subtilis* MF497446 isolated from the rhizosphere of wheat, which tolerated Cd up to a concentration of 18 mg L^−1^ [27]. In contrast, for *B. cereus* M4 (isolated from the rhizosphere of rice), it was reported that it tolerated a maximum Cd concentration of 1000 mg kg^−1^ [28]. Like what was found in this study for *Citrobacter* sp. 101a and *Klebsiella* sp. 76b, for bacteria of the genera *Citrobacter* and *Klebsiella* (isolated from agricultural soils irrigated with wastewater), a MIC of 0.5–2 mM (equivalent to 56–224 mg L^−1^) and 1.25–1.75 mM (equivalent to 140–196 mg L^−1^), respectively, has been reported [29]. *Stenotrophomonas* sp. 67w showed a lower tolerance to Cd compared with *Stenotrophomonas* sp. CD02 strain (isolated from an agricultural soil contaminated with heavy metals), which was able to grow at a concentration of 4 mM (equivalent to 450 mg L^−1^) [30] or the strain *Stenotrophomonas* sp CIK‐517Y (isolated from the corn rhizosphere in a crop irrigated with water contaminated by effluents from a tannery), which grew in the presence of 500 mg L^−1^ of Cd [31]. The difference in tolerance to Cd could be related to the adaptation of the bacteria to survive a contamination event [32].

The MIC reflects bacterial tolerance to Cd, a key determinant for soil bioremediation feasibility. In Colombian cacao‐growing soils, field studies report Cd concentrations up to 47 mg kg^−1^ [19], with this study′s target region peaking at 21.29 mg kg^−1^ [15]. These concentrations are far below the MIC range of the evaluated strains (MIC from 90 to 300 mg L^−1^, Figure 1), confirming their suitability for Cd remediation in these agricultural systems.

### 3.2. Cd Removal in Solution by Ureolytic Bacteria

**Figure 2:**
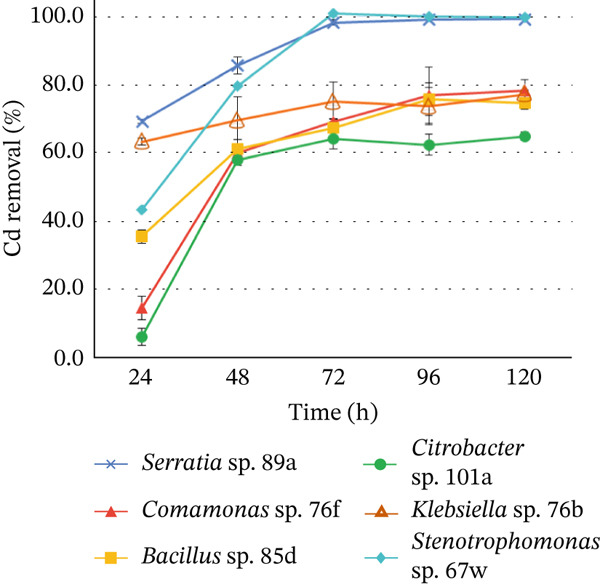
Cd removal (%) of ureolytic bacteria in LB medium supplemented with urea (20 g L^−1^), Ca (1.2 g L^−1^), and Cd (60 mg L^−1^) during 120 h of incubation at 30°C and 120 rpm. Error bars indicate SD (*n* = 3).

*Stenotrophomonas* sp. 67w and *Serratia* sp. 89a demonstrated exceptional performance, achieving > 99% Cd removal at 72 and 96 h, respectively (Figure 2). *Bacillus* sp. 85d reached 75.7% (±4.8%) after 96 h, whereas *Comamonas* sp. 76f and *Klebsiella* sp. 76b attained 78.3% (±0.16%) and 77.2% (±4.3%), respectively, at 120 h. *Citrobacter* sp. 101a showed the lowest efficacy (64.9*%* ± 1.2*%*). Extending the reaction time resulted in higher metal removal percentages for each bacterial strain evaluated. No visible precipitate formation was observed in the abiotic control throughout the experimental period, and Cd concentrations remained unchanged as determined by AAS.

In general, it was observed that Cd removal varied between 64.9% and 99.8% after 120 h of incubation and occurs mainly in 48 h (> 50%) (Figure 2), as found for the strains *Serratia* sp. 4.1a and 5b under similar experimental conditions [22]. In our study, Cd removal reached a maximum at 72 h for *Stenotrophomonas* sp. 67w, *Serratia* sp. 89a, *Klebsiella* sp. 76b, and *Citrobacter* sp. 101a, and at 96 h for *Bacillus* sp. 85d and *Comamonas* sp. 76f. In another study, for *Bacillus* sp. GZ‐22 strain with MICP activity, the highest Cd removal was reported at 10 h, starting from an initial Cd concentration six times lower than that used in our research [14]. This is in line with that reported by Qiao et al. [33], where the time to reach maximum removal increases with increasing initial Cd concentration. The authors reported a maximum removal at 0.5 h for Cd concentrations < 80 mg L^−1^ and at 6 h for 160 mg L^−1^. These results, together with other reports [14, 22, 33], indicate that Cd removal in solution increases with metal exposure time. However, metal removal eventually reached a plateau. The differences observed for Cd removal with respect to time, evidences the efficiency of each bacterium, where *Serratia* sp. 89a and *Stenotrophomonas* sp. 67w stand out with the highest removal in a shorter time.

### 3.3. Removal of Cd in Bioprecipitation and Biosorption Assays

In the bioprecipitation assay, the removal percentages varied between 59.9% and 85.2% (Table 1). *Serratia* sp. 89a showed the highest removal percentage, whereas the lowest corresponded to *Citrobacter* sp. 101a. Regarding the biosorption assay (where carbonate precipitation is not induced), it was observed that the removal varied between 25.0% and 47.0%, being lower than that found for the MICP assay. Interestingly, the bacteria with the highest and lowest Cd removal in the biosorption assay, *Klebsiella* sp. 76b and *Comamonas* sp. 101a, respectively, were different from those found for the bioprecipitation assay. Comparing both experiments, the greatest variation in Cd removal was for *Comamonas* sp. 76f, with a decrease of 50.5%. Like our results, it was reported that Cd removal through MICP was 11% higher than that observed by biosorption for *Bacillus* sp. GZ‐22 [14]. On the other hand, a similar biosorption was reported for *S. marcescens* NCIM 2919 and *E. cloacae* EMB19 for which, respectively, 7% and 12% Cd removal was observed from an initial concentration of 5 mg L^−1^ after 96 h [34]. During the immobilization of Cu, Pb, and Zn with the ureolytic bacterium *B. intermedia* TSBOI, it was reported that the metals concentration in solution decreased in the presence of only cells or cell debris, which was attributed by the authors to a biosorption process [35].

**Table 1:** Comparison of Cd removal percentages by ureolytic bacteria during bioprecipitation and biosorption assays.

Bacteria	Cd removed (%) ± SD in bioprecipitation assay	Cd removed (%) ± SD in biosorption assay
*Serratia* sp. 89a	85.2 ± 7.9∗	39.1 ± 0.9
*Comamonas* sp. 76f	72.7 ± 3.4∗	25.0 ± 1.1
*Bacillus* sp. 85d	63.7 ± 8.3∗	28.4 ± 4.7
*Citrobacter* sp. 101a	59.9 ± 1.4∗	33.0 ± 4.7
*Klebsiella* sp. 76b	61.6 ± 8.6∗	47.0 ± 1.2
*Stenotrophomonas* sp*. 67w*	80.6 ± 2.6∗	40.4 ± 3.3

*Note:* SD (standard deviation, *n* = 3). Asterisk “∗” indicates statistically significant differences within each strain by *t*‐test.

From the Cd removed in both assays, it can be estimated that the Cd bioprecipitated (Equation 1) for each bacterium was: 63.9% for *Comamonas* sp. 76f; 55.5% for *Bacillus* sp. 85d; 48.4% for *Serratia* sp. 89a; 42.5% for *Stenotrophomonas* sp. 67w; 35.7% for *Klebsiella* sp. 76b; and 34.6% for *Citrobacter* sp. 101a. This shows that for *Bacillus* sp. 85d and *Comamonas* sp. 76f, more than 50% of the Cd removed was by bioprecipitation. These results indicate that, for some bacteria, biosorption contributes mainly to Cd removal.

### 3.4. Sequential Extraction of Bioadsorbed, Bioprecipitated, and Bioaccumulated Cd in Bioprecipitation Assays

**Figure 3:**
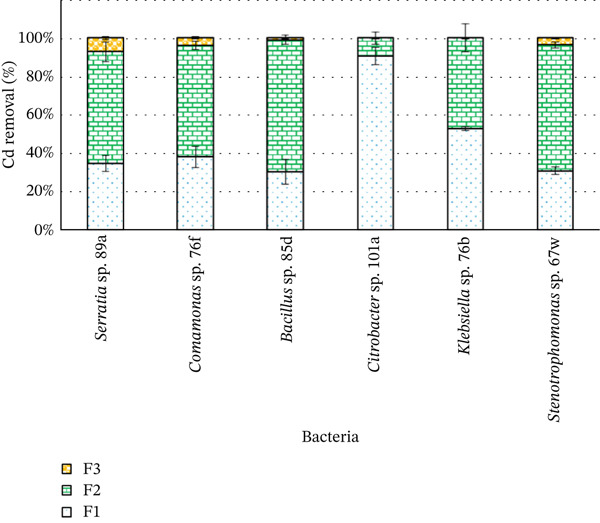
Removal of Cd (%) associated with each fraction in the bioprecipitation assay. F1 (dotted), F2 (horizontal brick), and F3 (sphere) fractions were defined as follows: F1 (EDTA‐extractable fraction, corresponding to bioadsorbed Cd), F2 (HCl‐extractable fraction, corresponding to bioprecipitated Cd), and F3 (aqua regia‐extractable fraction, corresponding to bioaccumulated Cd). To enable comparison among fractions, total Cd removal percentages were normalized to 100%. The error bars correspond to 1 standard deviation (*n* = 3).

From the sequential extractions of Cd carried out on the biomass‐precipitate mixture after applying the MICP process, similarities were observed in the distribution of the Cd removed when comparing the different bacteria (Figure 3). In general, it can be mentioned that Cd is mainly associated with F2 fraction, which corresponds to the Cd extracted with HCl (operationally defined as bioprecipitated Cd). The percentages of Cd removal associated with the bioprecipitation phenomenon (F2) for *Serratia* sp. 89a (54.5%), *Comamonas* sp. 76f (58.3%), *Bacillus* sp. 85d (65.1%), and *Stenotrophomonas* sp. 67w (60.4%) were greater than 50% of the total Cd removed. However, in the case of *Citrobacter* sp. 101a and *Klebsiella* sp. 76b, the main fraction was F1 (EDTA‐extracted Cd: bioadsorbed Cd), corresponding to 92.8% and 56.8%, respectively. Regarding the Cd extracted with aqua regia (bioaccumulated Cd, F3), the highest removal percentage was for *Serratia* sp. 89a with a value of 6.6%, indicating that bioaccumulation has a low contribution to Cd removal. These results are like those found for *Pseudochrobactrum* sp. DL‐1, where the metal distribution analysis indicated that Cd was removed mainly by bioprecipitation (49.27%), followed by bioadsorption (36.35%), and a smaller amount was accumulated (3.97%) within the bacterial cells [11].

These results highlight notable strain‐specific differences in Cd removal attributed to bioprecipitation, bioadsorption, and bioaccumulation among various bacterial genera. These differences can be explained by intrinsic bacterial properties such as cell wall composition, surface charge, extracellular polymeric substances, and enzyme activities like urease activity. Bacterial cell walls vary between genera and species, impacting their interaction with metals. Gram‐negative bacteria (e.g., *Serratia*, *Comamonas*, *Citrobacter*, and *Klebsiella*) have thinner peptidoglycan layers and possess an outer membrane rich in lipopolysaccharides, which carry more negative charges compared with gram‐positive bacteria [36]. These features can enhance electrostatic attraction and the availability of binding sites for Cd ions, promoting bioadsorption processes. Intracellular uptake (bioaccumulation) is limited for most strains (as shown by low F3 values), likely due to active metal efflux systems or lower cellular permeability to Cd, which prevents accumulation and reduces toxicity [37].

### 3.5. Sequential Extraction of Bioadsorbed, Bioprecipitated, and Bioaccumulated Cd in Biosorption Assays

**Figure 4:**
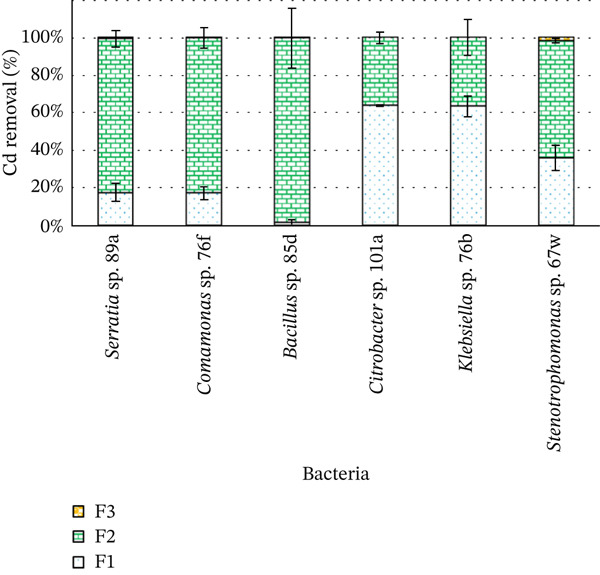
Removal of Cd (%) associated with each fraction in the biosorption assay. F1 (dotted), F2 (horizontal brick), and F3 (sphere) fractions were defined as follows: F1 (EDTA‐extractable fraction, corresponding to bioadsorbed Cd), F2 (HCl‐extractable fraction, corresponding to bioadsorbed Cd), and F3 (aqua regia‐extractable fraction, corresponding to bioaccumulated Cd). To enable comparison among fractions, total Cd removal percentages were normalized to 100%. The error bars correspond to 1 standard deviation (*n* = 3).

To further evaluate the selectivity of the sequential extraction procedure for differentiating bioadsorbed and bioprecipitated Cd, the same sequential extraction was applied to the biosorption assay, where it was expected that Cd would not be associated with F2 because carbonate precipitation was not induced in this assay. Surprisingly, the results were similar when comparing the bioprecipitation and biosorption assays, since in the latter, the F2 fraction (extracted with HCl) was the main one for *Serratia* sp. 89a, *Comamonas* sp. 76f, *Bacillus* sp. 85d, and *Stenotrophomonas* sp. 67w (Figure 4). On the other hand, percentages between 1.6% and 63.8% were obtained for the fraction extracted with EDTA (F1). Since carbonate precipitation was not induced in this medium, Fractions F1 and F2 correspond to bioadsorbed Cd. The fact that Cd was found associated with F2 indicates that the EDTA solution did not extract all the bioadsorbed Cd. Thus, in the bioprecipitation assay, the bioadsorbed Cd associated with fraction F1 is probably underestimated.

These findings highlight an important limitation of the sequential extraction protocol used in this study, since the HCl‐extractable fraction (F2) cannot be considered exclusively representative of bioprecipitated Cd. Instead, the results suggest that part of the Cd associated with F2 may also correspond to strongly bound bioadsorbed Cd that was not removed during the EDTA extraction step. Therefore, the operational definition of F2 should be interpreted with caution, as overlap between bioadsorbed and bioprecipitated fractions may occur.

The results found can be explained considering the capacity of different solutions to extract bioadsorbed Cd. It has been reported that HCl has a greater capacity than EDTA to extract bioadsorbed Cd [20, 21]. Furthermore, differences in the metal–bacteria interaction (e.g., ion exchange, complexation, or physical entrapment) affect the metal desorption capacity of different extracting solutions [20]. These authors also reported a methodology to determine precipitated, bioadsorbed, and bioaccumulated Cd, which was based on the initial separation of carbonated precipitates and biomass by centrifugation due to differences in density [11]. However, in the present study this separation was not possible, probably due to the aggregation of biomass or the tendency of bacteria to adhere to mineral surfaces [38].

The evaluation of the potential for Cd bioremediation showed that strain *Stenotrophomonas* sp. 67w stood out by achieving a removal greater than 99% in 72 h (Figure 2). In addition, *Bacillus* sp. 85d presented a high percentage of bioprecipitated Cd (Table 1, Figure 3). Thus, these two bacteria were selected to analyze their precipitates.

### 3.6. Bioprecipitates Analysis

**Figure 5:**
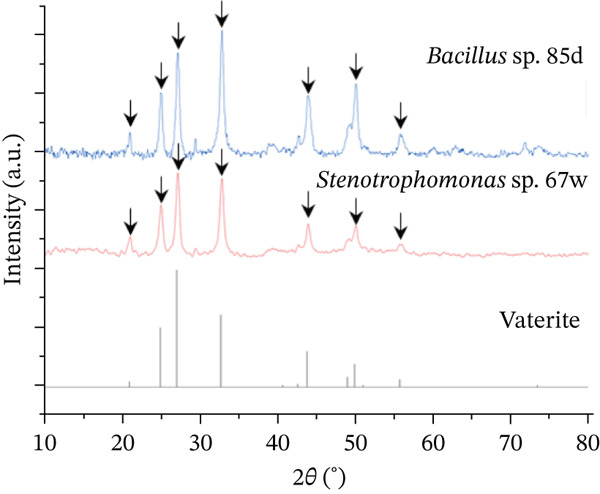
Diffractogram of *Bacillus* sp. 85d and *Stenotrophomonas* sp. 67w precipitates. The reference peaks of vaterite were taken from the database of the X′Pert HighScore Plus software. Arrows indicate characteristic peaks of vaterite.

XRD analyses showed the presence of vaterite for precipitates of *Bacillus* sp. 85d and *Stenotrophomonas* sp. 67w, due to the characteristic peaks observed around 20°, 24°, 27°, 32°, 43°, 50°, and 55° of 2*θ* (Figure 5). Vaterite is one of the polymorphs of calcium carbonate commonly found in assays using bacteria with MICP activity [39], despite being less stable than calcite [40]. It has been pointed out that the calcium carbonate polymorphs (calcite, aragonite, or vaterite) formed in MICP assays are related to the bacteria and the composition of the medium used [41], although there is no consensus on these aspects. In a study using different Ca sources (calcium chloride, calcium acetate, calcium nitrate, and calcium lactate), it was reported that calcium chloride favors the formation of vaterite [42], which is consistent with what was found in our study. However, the use of this calcium source does not imply the exclusive formation of this polymorph. In the case of *Sporosarcina pasteurii* KCTC 3558, using different calcium sources, including calcium chloride, the formation of precipitates corresponding to mixtures of vaterite and calcite was observed [43]. Wen et al. [44] conducted a study using two systems to induce carbonate precipitation, one in which *S. pasteurii* (ATCC 11859) was used and another with commercial urease. The authors reported that the system with the bacteria induces the formation of vaterite while the system with the commercial enzyme induces the formation of calcite. On the other hand, during the MICP process it was found that the bacterial cells of *Bacillus cereus* LV‐1 did not promote the formation of vaterite, indicating that other factors, such as pH, influence to a greater extent the formation of this polymorph [45]. It has also been pointed out that the polymorph obtained could be associated with differences in the urease activity of the bacteria [46]. This indicates that further studies are needed to explain the production of vaterite by *Bacillus* sp. 85d and *Stenotrophomonas* sp. 67w.

**Figure 6:**
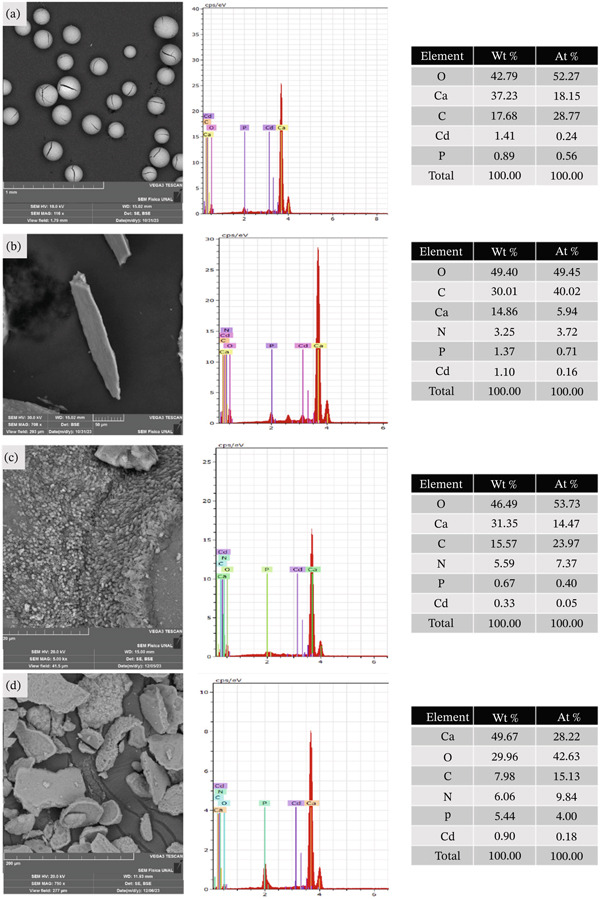
SEM micrographs and their respective EDX spectra for the precipitates of (a–b) *Bacillus* sp. 85d and (c–d) *Stenotrophomonas* sp. 67w obtained after 15 days in LB medium supplemented with urea (20 g L^−1^), Ca (1.2 g L^−1^), and Cd (60 mg L^−1^) at 30°C and 120 rpm. Wt %, weight percentage; At %, atomic percentage.

SEM analysis showed different morphologies in the precipitates (Figure 6) with sphere‐ and needle‐like precipitates for *Bacillus* sp. 85d (Figure 6a,b), whereas the presence of calcified bacteria was observed for *Stenotrophomonas* sp. 67w (Figure 6c). Other studies reported the formation of spherical‐ [11] and needle‐like [47] precipitates after MICP process. The presence of spherical vaterite [48] agrees with the results obtained by XRD. However, the needle morphology corresponding to aragonite [49] was not observed in the diffractogram, probably due to a low proportion in the precipitates obtained. Regarding the EDX analysis, the results indicated the presence of Cd in the precipitates. Cd presented mass percentages of 1.41% and 1.10% (Figure 6a,b) in the case of *Bacillus* sp. 85d, and 0.33% and 0.90% (Figure 6c,d) for *Stenotrophomonas* sp. 67w. The analysis also showed the presence of N and P that may correspond to exopolymeric substances [33] or calcified bacteria [50], which would also explain why the proportion of C, Ca, and O differs from that expected for CaCO_3_ [11]. This is consistent with the calcified bacteria observed by SEM (Figure 6c).

Based on the quantification of Cd in precipitates by atomic absorption spectrophotometry, it was determined that the needle‐shaped precipitates of *Bacillus* sp. 85d had a mass Cd percentage of 0.57%, which is higher than that found for the spherical precipitates (0.32%), and the precipitates of *Stenotrophomonas* sp. 67w presented lower Cd (0.19%) compared with *Bacillus* sp. 85d. These results confirm that the MICP process allows the mineral precipitation of Cd. Overall, the results from the bioprecipitation and biosorption assays, along with precipitate analyses, demonstrate that three distinct approaches can be employed to determine the percentage of bioprecipitated Cd:

a. Bioprecipitated Cd can be estimated by calculating the difference in Cd removal between the bioprecipitation and biosorption assays. Specifically, subtracting the amount of bioadsorbed Cd from the total Cd removed in the bioprecipitation assay provides an estimate of the precipitated Cd.
b. Sequential extraction using different solutions allows for the estimation of how much Cd is bioadsorbed, bioprecipitated, and bioaccumulated. We observed that EDTA solution only partially extracted bioadsorbed Cd, highlighting the need for further optimization of extraction protocols. Additionally, to validate extraction efficiency, alternative methods such as isotopic labeling should be performed.
c. Cd in carbonate precipitates was quantified via AAS following dissolution in HCl. This was complemented by XRD and SEM‐EDX analyses to confirm the presence of carbonates and Cd incorporation via coprecipitation. Precipitate characterization revealed Cd coprecipitation with the calcium carbonate (CaCO_3_) polymorph vaterite. Quantification of Cd in precipitates by AAS improves previous works focusing only on analysis of operationally defined fractions extracted using diverse solutions.

## 4. Conclusions

The ureolytic bacterial strains achieved Cd removal rates of 64.9%–99.8% within 120 h in a media containing 60 mg L^−1^ of Cd. Furthermore, MIC assays showed that these strains tolerate Cd levels ranging from 90 to 300 mg L^−1^, significantly higher than those found in agricultural soils, such as those used for cacao cultivation. These results highlight the robust survival and remediation capabilities of those bacteria.

Our study revealed that bioadsorption contributes significantly to Cd removal during MICP. Importantly, four of the six bacterial strains (*Serratia* sp. 89a, *Citrobacter* sp. 101a, *Klebsiella* sp. 76b, and *Stenotrophomonas* sp. 67w) immobilized > 50% of Cd via bioadsorption, whereas bioprecipitation accounted for 34.6%–63.9% of total removal. *Bacillus* sp. 85d and *Comamonas* sp. 76f primarily removed Cd through bioprecipitation (> 50%). Precipitates analysis confirmed Cd coprecipitation within a vaterite (CaCO_3_) matrix.

These results emphasize that assessing the MICP potential of ureolytic bacteria should extend beyond Cd removal percentages to include quantification of bioprecipitated and bioadsorbed Cd. This study provides a methodological framework to improve the understanding and application of the MICP biotechnology.

The demonstrated ability of ureolytic bacteria to tolerate and immobilize high Cd concentrations highlights their potential for bioremediation; however, further studies under soil conditions are required to validate their effectiveness in contaminated agricultural systems. MICP could help stabilize Cd in less bioavailable forms. The dual mechanisms identified (biosorption and bioprecipitation as Cd–CaCO_3_) may enhance remediation efficiency since they provide a framework to select bacterial strains based on their dominant immobilization pathways, optimizing field‐scale applications considering long term stability.

## Funding

This study was supported by Dirección de Investigación y Extensión sede Bogotá (48328, 48335, 57665, 62196) and Ministerio de Ciencia, Tecnología e Innovación (MinCiencias), Colombia (110180863795/CT‐190‐2019).

## Conflicts of Interest

The authors declare no conflicts of interest.

## Supporting information


**Supporting Information** Additional supporting information can be found online in the Supporting Information section. The supporting material presents the protocol for the sequential extraction of bioadsorbed, bioprecipitated, and bioaccumulated Cd in bioprecipitation and biosorption experiments. Figure S1 shows a graphical scheme for these procedures.

## Data Availability

Data are available on request from the authors.
