# Supplementary material for: Ureolytic Rhizobacteria for Cadmium Remediation: Contribution of Bioadsorption and Bioaccumulation During Microbially Induced Carbonate Precipitation in Liquid Medium Assays
Source: Int J Microbiol. 2026 Aug 3;2026:9681937. doi: 10.1155/ijm/9681937 (PMC13434221; doi:10.1155/ijm/9681937)
Supplement: Supplementary file 1 [file IJM-2026-9681937-s001.docx]

**Supplementary material for article:**

**Ureolytic rhizobacteria for cadmium remediation: contribution of bioadsorption and bioaccumulation during microbially induced carbonate precipitation in liquid medium assays**

Carlos A. Adarme-Duran^1,2^, Elianna Castillo^2^, Pedro F. B. Brandão^2,*^

^1^Universidad Nacional de Colombia - sede Bogotá – Facultad de Ciencias – Instituto de Biotecnología - IBUN – Carrera 30 # 45-03 – Bogotá – Colombia

^2^Universidad Nacional de Colombia - sede Bogotá – Facultad de Ciencias – Departamento de Química – Grupo de Estudios para la Remediación y Mitigación de Impactos Negativos al Ambiente (GERMINA) – Carrera 30 # 45-03 – Bogotá – Colombia

*Corresponding author: pfdeb@unal.edu.co

Below is presented the protocol for the sequential extraction of bioadsorbed, bioprecipitated and bioaccumulated Cd in bioprecipitation and biosorption experiments (Shen et al., 2021; Sheng et al., 2022; Vishan et al., 2019). Figure S1, shows a graphical scheme for these procedures.

1) After the incubation time of the bioprecipitation and biosorption experiments, all the respective culture medium (10 mL) was centrifuged at 8500 rpm for 10 min. The supernatant was collected and used to determine the total cadmium removed from solution for each experiment (**Total Cd removed**). The pellet was washed three times with deionized water (2 mL). The pellet obtained corresponds to a mixture of precipitates and bacterial biomass.

2) The pellet was resuspended in 2 mL of EDTA (20 mM, pH 7) and gently agitated for 30 seconds. This mixture was centrifuged at 8500 rpm for 10 min to collect the supernatant. The pellet was washed three times with deionized water (2 mL), and the washing liquid was mixed with the latter collected supernatant. The collected supernatants were used to determine the bioadsorbed cadmium (**Cd bioadsorbed – F1**).

3) Two mL of HCl (1,2 M) was added to the previously treated biomass and gently agitated to dissolve the carbonate precipitates. After centrifugation (8500 rpm for 10 min), the supernatant was collected and used to determine the bioprecipitated cadmium (**Cd bioprecipitated – F2**). After these procedures, the pellet was considered as bacterial biomass only.

4) The latter bacterial biomass was treated with 8 mL aqua regia (3 HCl : 1 HNO_3_) and digested using the following temperature program: an increase from ambient temperature (approx. 20 °C) to 120 °C in 30 min, and the temperature was hold at 120 °C for 120 min. The obtained solution was considered as the cadmium accumulated in cells (**Cd bioaccumulated – F3**).

**NOTE 1:** F1 and F2 were treated with 1 mL of aqua regia. All fractions were filtered (0.22 µm) and made up to volume (10 mL) using HNO_3_ (0,5 % v/v).

**NOTE 2:** Cd was determined by flame atomic absorption spectroscopy – AAS (ContrAA 700 - High-Resolution Continuum Source, Analytik Jena, Alemania). See manuscript for details.

**Figure S1.** Graphical protocol for the sequential extraction of bioadsorbed, bioprecipitated and bioaccumulated Cd obtained in bioprecipitation and biosorption experiments.

**References**

**Shen, L., Chen, R., Wang, J., Fan, L., Cui, L., Zhang, Y., Cheng, J., Wu, X., Li, J., & Zeng, W. (2021). Biosorption behavior and mechanism of cadmium from aqueous solutions by: *Synechocystis* sp. PCC6803. RSC Advances, 11(30), 18637–18650. https://doi.org/10.1039/d1ra02366g**

**Sheng, M., Peng, D., Luo, S., Ni, T., Luo, H., Zhang, R., Wen, Y., & Xu, H. (2022). Micro-dynamic process of cadmium removal by microbial induced carbonate precipitation. Environmental Pollution, 308. https://doi.org/10.1016/j.envpol.2022.119585**

**Vishan, I., Saha, B., Sivaprakasam, S., & Kalamdhad, A. (2019). Evaluation of Cd(II) biosorption in aqueous solution by using lyophilized biomass of novel bacterial strain *Bacillus badius* AK: Biosorption kinetics, thermodynamics and mechanism. Environmental Technology and Innovation, 14. https://doi.org/10.1016/j.eti.2019.100323**
